# In Vitro Osteogenic Properties of Two Dental Implant Surfaces

**DOI:** 10.1155/2012/181024

**Published:** 2012-10-15

**Authors:** Marta Monjo, Christiane Petzold, Joana Maria Ramis, Staale Petter Lyngstadaas, Jan Eirik Ellingsen

**Affiliations:** ^1^Department of Fundamental Biology and Health Sciences, Research Institute on Health Sciences (IUNICS), University of Balearic Islands, E-07122 Palma de Mallorca, Spain; ^2^Department of Biomaterials, University of Oslo, 0317 Oslo, Norway; ^3^Oral Research Laboratory, Institute for Clinical Dentistry, University of Oslo, 0317 Oslo, Norway

## Abstract

Current dental implant research aims at understanding the biological basis for successful implant therapy. The aim of the study was to perform a full characterization of the effect of two commercial titanium (Ti) surfaces, OsseoSpeed and TiOblast, on the behaviour of mouse preosteoblast MC3T3-E1 cells. The effect of these Ti surfaces was compared with tissue culture plastic (TCP). In vitro experiments were performed to evaluate cytotoxicity, cell morphology and proliferation, alkaline phosphatase activity, gene expression, and release of a wide array of osteoblast markers. No differences were observed on cell viability and cell proliferation. However, changes were observed in cell shape after 2 days, with a more branched morphology on OsseoSpeed compared to TiOblast. Moreover, OsseoSpeed surface increased BMP-2 secretion after 2 days, and this was followed by increased IGF-I, BSP, and osterix gene expression and mineralization compared to TiOblast after 14 days. As compared to the gold standard TCP, both Ti surfaces induced higher osteocalcin and OPG release than TCP and differential temporal gene expression of osteogenic markers. The results demonstrate that the gain of using OsseoSpeed surface is an improved osteoblast differentiation and mineralization, without additional effects on cell viability or proliferation.

## 1. Introduction

Current dental implant research aims at developing of innovative surfaces able to promote a more favourable biological response to the implant material at the bone-implant interface and to accelerate osseointegration [1]. It has largely been demonstrated that rough surfaces present an increased bone fixation and bone-to-implant contact compared to smooth surfaces [2–4]. In addition to surface topography, the chemical properties of implant surfaces also play an important role in promoting osseointegration [5]. Modification of titanium implants using hydrogen fluoride at low concentrations results in the formation of nanostructures along the titanium surface as well as the incorporation of small amounts of fluoride into the crystal structure of the superficial layer of the implant [1, 6], thereby, modifying both, surface topography and surface chemistry. 

In vitro experiments have shown that fluoride-modified titanium implants stimulate osteoblast differentiation in different cell models [7–10], enhance cell osteoblastic adhesion and expression of bone-specific mRNA [8, 11], increase cell viability [11], improve the initial cell response to the implant [12], and augment the thrombogenic properties of titanium, promoting fibrinogen activation and rapid coagulation [13]. In vivo, fluoride-modified titanium implants enhance interfacial bone formation [8], create a firmer bone anchorage [14], augment the amount of new bone formation in the voids and bone-to-implant contact [15], improve biomechanical properties due to a more mature and mineralized interfacial bone matrix [16], and increase implant osseointegration in osteoporotic bone [17]. 

The aim of the present study was to examine the in vitro bone response of mouse preosteoblast MC3T3-E1 cells to two commercial Ti surfaces, OsseoSpeed and TiOblast, and to validate the claimed higher bone response of the new generation surface (OsseoSpeed) compared to its respective predecessor (TiOblast). The osteoblast response to these Ti surfaces was also compared with tissue culture plastic (TCP), which is normally considered the gold standard for tissue culture. OsseoSpeed is a further development of the moderately roughened (grit blasted with titanium dioxide particles) titanium surface TiOblast. OsseoSpeed has been reported to gain its additional surface characteristics via a chemical (fluoride) treatment and a slight topographic modification of the TiOblast surface [6, 14]. 

## 2. Materials and Methods

### 2.1. Implants and Treatments

Test implants used were all made of grade 2 titanium, with a diameter of 6.25 mm and a height of 1.95 mm. The test surface was blasted with titanium dioxide (TiO_2_) particles (TiOblast) to create a microrough surface. According to the manufacturer, fluoride modified implants (OsseoSpeed) went through an additional cleaning process including diluted HF. The blasted-only implants served as control. Implants were premounted on the carriers, inserted individually in sealed containers, and sterilized by *β*-irradiation (AstraTech AB, Mölndal, Sweden). 

### 2.2. Roughness Analysis

After surface treatments, the surface roughness of one sample per group was measured with a blue light profilometer (PL*μ* 2300, Sensofar, Terassa, Spain). Three areas were imaged per surface at 50x magnification (254 × 191 *μ*m^2^), and surface parameters were calculated after levelling the images by rotation with the program SensoMap Plus 4.1 (SARL Digital Surf, Besançon, France) and application of a Gaussian filter (50 × 50 *μ*m) to remove underlying waviness. Topographical changes were as follows: average height deviation from the mean plan (*S*_*a*_), surface skewness (*S*_sk_), surface kurtosis (*S*_ku_), and core fluid retention index (*S*_ci_) were recorded to quantify surface differences among the TiOblast and the OsseoSpeed groups. 

### 2.3. Cell Culture

The murine osteoblast cell line MC3T3-E1 (DSMZ, Braunschweig, Germany) was used as in vitro model. Cells were routinely cultured at 37°C in a humidified atmosphere of 5% CO_2_ and maintained in alpha-MEM (PAA Laboratories GmbH, Austria) supplemented with 10% fetal calf serum (PAA Laboratories GmbH, Austria) and 50 IU penicillin/mL and 50 *μ*g streptomycin/mL (Sigma, St. Louis, MO, USA). Cells were subcultured 1 : 4 before reaching confluence using PBS (PAA Laboratories GmbH, Austria) and trypsin/EDTA (Sigma, St. Louis, MO, USA). To test the different surface modification of titanium implants (TiOblast and OsseoSpeed), coins were placed in a 96-well plate (with a diameter size per well of 6.5 mm), and 10^4^ cells were seeded on each coin. The same number of cells was cultured in parallel in plastic (TCP) in all the experiments. 

### 2.4. Cell Viability

LDH activity was used as an index of cytotoxicity in the culture media. After 48 h, 7, and 14 days, the culture media was collected, centrifuged at 500 ×g for 5 min at 4°C, and the supernatant was stored at 4°C. LDH activity was determined spectrophotometrically according to the manufacturer's kit instructions (Cytotoxicity Detection kit, Roche Diagnostics, Mannheim, Germany) and presented relative to the LDH activity in the medium of cells cultured on TCP for 2 days, which was set as 100%.

### 2.5. DAPI Staining and Cell Counting

MC3T3-E1 cells were seeded on the coins modified according to the protocol described above. The MC3T3-E1-cell layers were washed twice with PBS after the respective culture time and fixed in a 4% formaldehyde solution in PBS for 30 min. Subsequently, the samples were washed again with PBS and mounted with a DAPI-containing mounting medium (ProLong Gold antifade reagent with DAPI, Invitrogen Ltd, Paisley, UK) according to their protocol. The samples were stored in dark at −20°C until analysis by fluorescence microscopy (Leica DM RBE, Leica Microsystems, Wetzlar, Germany) with connected digital camera (Olympus DM50, Olympus Europe, Hamburg, Germany). Afterwards, nuclei were counted with the ImageJ software (http://rsbweb.nih.gov/ij/). Four implants were used per treatment and time point, and in each implant three different fields were analysed, giving the number of cells per *μ*m^2^.

### 2.6. SEM Analysis

SEM analyses were performed to study the morphology of MC3T3-E1 cells grown on the surface of both Ti surfaces. For this purpose, cells were first fixed in a formaldehyde solution at 4% in PBS for 30 min. Cell layers were dried in increasing concentrations of ethanol followed by critical point drying (E3000, Quorum Tech, Ashford, UK) and sputter coated with a thin layer of carbon (Cressington Carbon Coater 108/carbon A, Cressington Scientific Intruments Ltd., Watford, UK). SEM scans were taken (Philips XL 30 ESEM, FEI Electron Optics, Eindhoven, The Netherlands) to image the morphology of the cells attaching to the different surfaces. Pictures at 400x of magnification were taken after 2, 7, and 14 days and also at 2000x of magnification after 2 days.

### 2.7. Release of BMP-2 into the Cell Culture Media

Cell culture supernatants were analysed for BMP-2 release, secreted to the culture medium after 2 days of cell culture, using an enzyme-linked immunosorbent assay (ELISA). Aliquots from the culture media were centrifuged at 1800 rpm for 5 minutes at 4°C, and supernatants were used for BMP-2 determination following instructions described by the manufacturer (Quantikine Immunoassay, R&D Systems, Minneapolis, MN, USA). 

### 2.8. Isolation of Total RNA

Total RNA was isolated using a monophasic solution of phenol and guanidine isothiocyanate (Trizol, Invitrogen Life Technologies, Carlsbad, CA, USA), following the instructions of the manufacturer. RNA was quantified at 260 nm using a Nanodrop spectrophotometer (NanoDrop Technologies, Wilmington, DE, USA).

### 2.9. Real-Time RT-PCR

The same amount of total RNA (2 *μ*g) from each sample was reverse transcribed to cDNA at 42°C for 60 min in a final volume of 40 *μ*L, using iScript cDNA Synthesis kit (BioRad) that contains both oligo(dT) and random hexamers. Each cDNA was diluted 1/5, and aliquots were frozen (−20°C) until the PCR reactions were carried out. Real-time PCR was performed for two housekeeping genes: 18S ribosomal RNA (18S rRNA), glyceraldehyde-3-phosphate dehydrogenase (GAPDH), and thirteen target genes: alkaline phosphatase (ALP), bone sialoprotein (BSP), CD44, collagen type I (coll-I), distal-less homeobox 2 (Dlx2), hairy and enhancer of split 1 (Hes1), insulin growth factor-I (IGF-I), interleukin-6 (IL-6), osteoprotegerin (OPG), osterix (Osx), receptor activator of NFkappaB ligand (RANKL), Smad1, and Smad5. Oligonucleotide primer sequences used for the real-time RT-PCR, the length of the resulting amplicons and the GeneBank accession number, are shown in Table 1.

Real-time PCR was performed in the iCycler (BioRad) using SYBR green detection. Each reaction contained 5 *μ*L of cDNA, 500 nM of the sense and antisense specific primers (for all, except for collagen-I which was 300 nM), and 12.5 *μ*L of 2X iQ SYBR Green Supermix in a final volume of 25 *μ*L. The amplification program consisted of a preincubation step for denaturation of the template cDNA (3 min 95°C), followed by 40 cycles consisting of a denaturation step (15 s 95°C), an annealing step (15 s 60°C; for all, except for ALP which was 65°C), and an extension step (30 s 72°C). After each cycle, fluorescence was measured at 72°C. A negative control without cDNA template was run in each assay. Samples were run in duplicate. 

Real-time efficiencies were calculated from the given slopes in the iCycler software using serial dilutions, showing all the investigated transcripts high real-time PCR efficiency rates, and high linearity (*r* > 0.99) when different concentrations were used. PCR products were subjected to a melting curve analysis on the iCycler and subsequently 2% agarose/TAE gel electrophoresis to confirm amplification specificity. 

### 2.10. Alkaline Phosphatase Activity

An aliquot of 25 *μ*L of culture media was assayed in duplicate for alkaline phosphatase activity by measuring the cleavage of p-Nitrophenyl Phosphate (pNPP) (Sigma, St. Louis, MO, USA) in a soluble yellow end product which absorbs at 405 nm. A volume of 100 *μ*L of this substrate was used. The reaction was stopped after 30 min in dark with the addition of 50 *μ*L of 3 M sodium hydroxide. The absorbance of the stopped reaction was read at 405 nm. In parallel to the samples, a standard curve with calf intestinal alkaline phosphatase (CIAP, 1 U/*μ*L) (Promega, Madison, WI, USA) was constructed, by mixing 1 *μ*L from the stock CIAP with 5 mL of alkaline phosphatase buffer (1 : 5000 dilution) and then making 1 : 5 serial dilutions.

### 2.11. Luminex Analysis

Cell culture supernatants were analysed for OPG, osteocalcin, IL-6, TNF-*α*, and RANKL using the solid phase sandwich multiplex bead immunoassays (Mouse Bone Panel 1B LINCOplex kit and Mouse RANKL single-plex kit, Cat#MBN1B-41K and Cat#MBN-41K-1RANKL, Linco Research, St. Charles, MO, USA) according to the manufacturer's protocol. Multianalyte profiling was performed on the Luminex-100 analyser (Luminex Corporation, Austin, TX, USA).

### 2.12. Calcium Crystal Deposition Quantification

After 14 days of cell culture, the entire surface of TiOblast and OsseoSpeed titanium coins (*n* = 2) were examined for calcium deposition with a tabletop scanning electron microscope (SEM) (TM-1000, Hitachi, Tokyo, Japan). Cell layers were dried in increasing concentrations of ethanol followed by critical point drying (E3000, Quorum Tech, Ashford, UK) and sputter coated with a thin layer of carbon (Cressington Carbon Coater 108/carbon A, Cressington Scientific Intruments Ltd, Watford, UK). A 250x magnification was used to examine the shape, size, and number of calcium crystals. The composition of the crystals was analysed by energy dispersive X-ray spectroscopy (EDS unit, TM-1000, Hitachi, Tokyo, Japan).

### 2.13. Statistical Analysis

All data are presented as mean values ± SEM. Differences between groups were assessed by Student's *t*-test, using the program SPSS for Windows, version 17.0. Results were considered statistically significant at the *P* ≤ 0.05 level.

## 3. Results

### 3.1. Surface Topographic Characterization

Topographical analyses by blue-light profilometry (Table 2) revealed a significant increase of *S*_*a*_ for the OsseoSpeed surfaces compared to TiOblast. Surface skewness was negative for both groups, and they can be imagined as bearing surfaces with holes. The skewness was significantly increased for OsseoSpeed. Surface kurtosis was significantly higher for TiOblast, indicating a more rounded appearance of the TiOblast surfaces. The core fluid retention index was significantly higher for OsseoSpeed, in accordance with the *S*_*a*_ values and pointing to a better fluid retention for OsseoSpeed surfaces.

### 3.2. Cell Adhesion and Morphology

**Figure 1:**
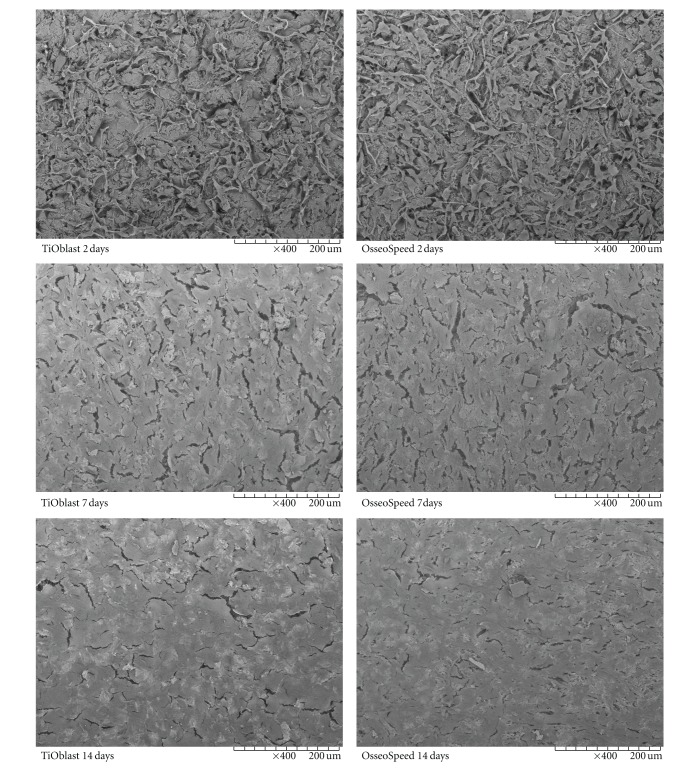
Scanning electron microscope images of MC3T3-E1 cells attached to each coin after 2, 7, and 14 days of cell culture. 400x magnification images are shown.

**Figure 2:**
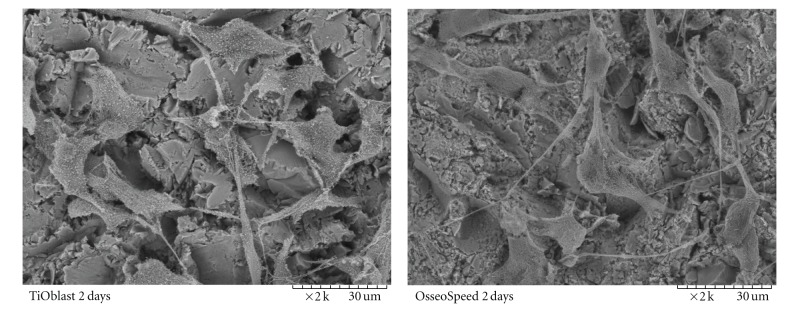
Detail of surface morphology and cell monolayer of MC3T3-E1 cells after 2 days by scanning electron microscope. 2000x magnification images are shown.

**Figure 3:**
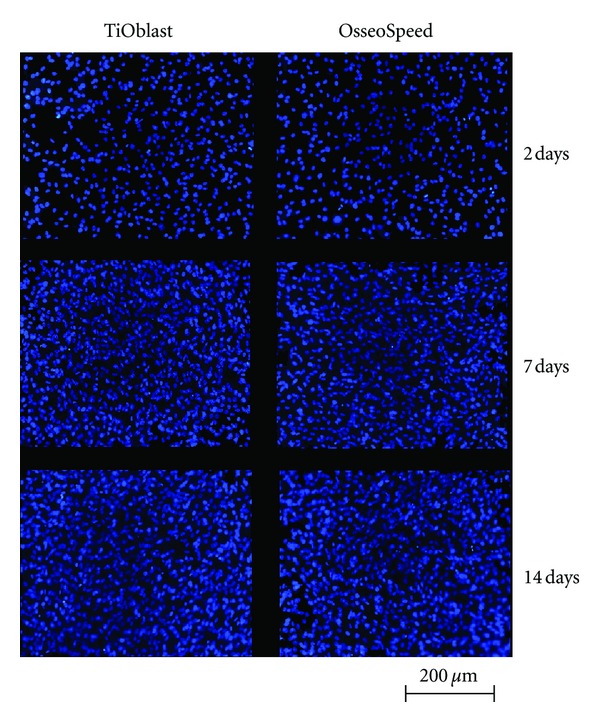
DAPI staining of the nuclei (20x) of the cell monolayer attached to each coin after 2, 7, and 14 days of cell culture.

Observation of the cell monolayer by SEM microscopy (Figures 1 and 2) and by fluorescence microscopy (Figure 3) confirmed that MC3T3-E1 cells attached well to both Ti surfaces, resulting in similar cell proliferation at the different time points investigated. Cell shape was examined in detail after 2 days. On OsseoSpeed surface, cells exhibited a more branched shape morphology compared to TiOblast.

### 3.3. Cell Viability, Proliferation, and RNA Content

**Figure 4:**
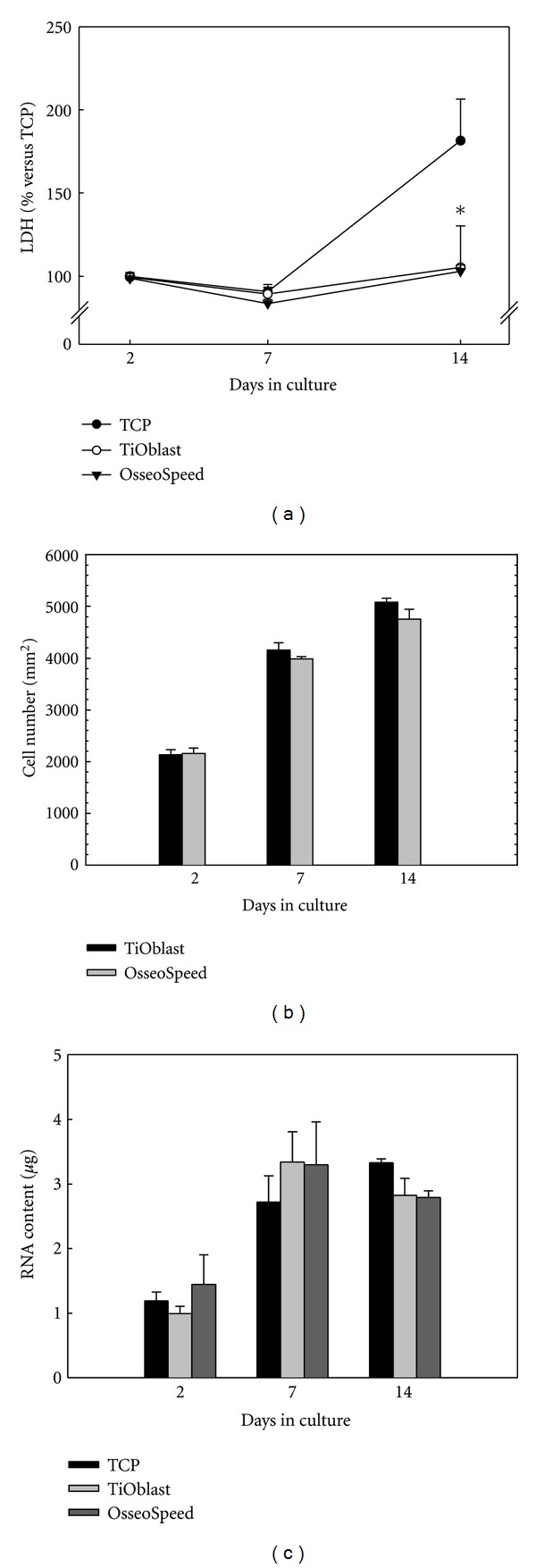
Cell viability, proliferation and RNA content of MC3T3-E1 cells (a) LDH activity measured from culture media collected after 2, 7 and 14 days of culture. Data expressed as a relative to the mean value of the TCP group at day 2, which was set as 100%. (b) Number of nuclei were counted with the ImageJ software and referred to the number of cells/mm^2^. (c) RNA content of cells attached after 2, 7 and 14 days of culture. Values represent the mean ± SEM. Student's *t*-test: *P ≤ 0.05 versus TCP; ^#^ *P* ≤ 0.05 versus TiOblast.

In order to determine the effect of the different Ti surfaces and TCP on the cell viability after short- and long-term cell culture on the titanium implants, the LDH activity in the culture media was measured after 2, 7, and 14 days (Figure 4(a)). No differences were found between the OsseoSpeed and TiOblast in the LDH activity at any of the days analysed. However, after 14 days of culture, cells cultured onto TCP showed significantly higher levels of LDH activity compared to cells cultured onto Ti implants. Counting of cells at the different time points using DAPI staining (Figure 4(b)), revealed no differences in cell proliferation between the two Ti surfaces. RNA content was quantified from cell monolayer after 2, 7, and 14 days of culture (Figure 4(c)). No differences were observed among the different groups, although RNA content increased from 2 to 7 days of cell culture.

### 3.4. BMP-2 Release

**Figure 5:**
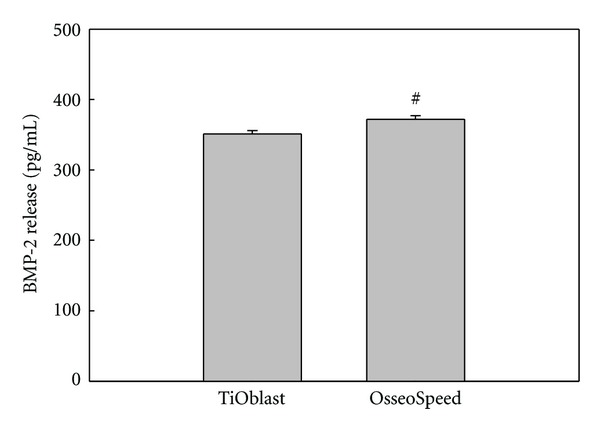
BMP-2 released to culture media after 2 days of culture. Values represent the mean ± SEM. Student's *t*-test: ^#^ *P* ≤ 0.05.

The potential to initiate osteogenic differentiation after 2 days of culture was investigated by measuring the BMP-2 release to the culture media using a BMP-2 immunoassay (Figure 5). OsseoSpeed titanium implants induced a significant higher release of BMP-2 compared to TiOblast. 

### 3.5. Gene Expression of Osteogenic Markers

**Figure 6:**
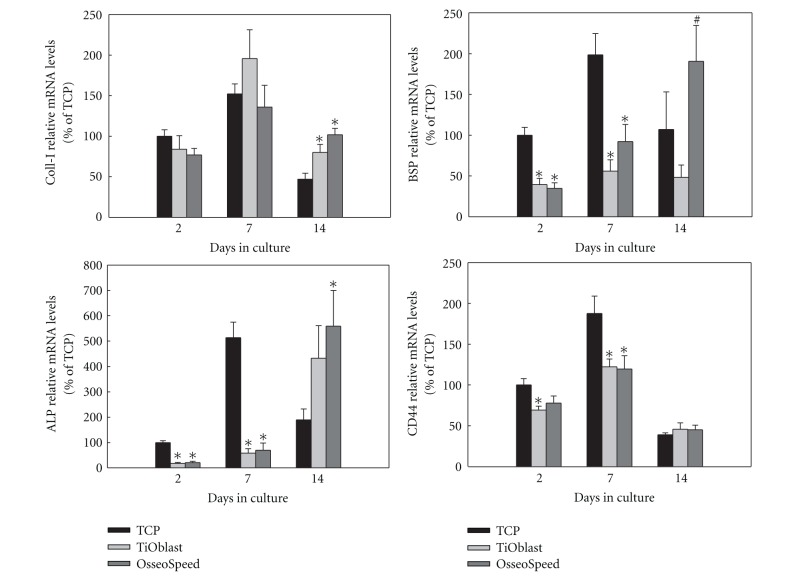
Relative mRNA levels of osteogenic markers (Coll-I, BSP, ALP, CD44) after 2, 7, and 14 days of culture. Ratios of target genes relative to housekeeping genes (GAPDH, B-actin) were expressed relative to the mean value of the TCP group at day 2, which was set as 100%. Values represent the mean ± SEM. Differences between groups (*n* = 6) were assessed by Student's *t*-test. ^#^ *P* ≤ 0.05 versus TiOblast; *P ≤ 0.05 versus TCP.

**Figure 7:**
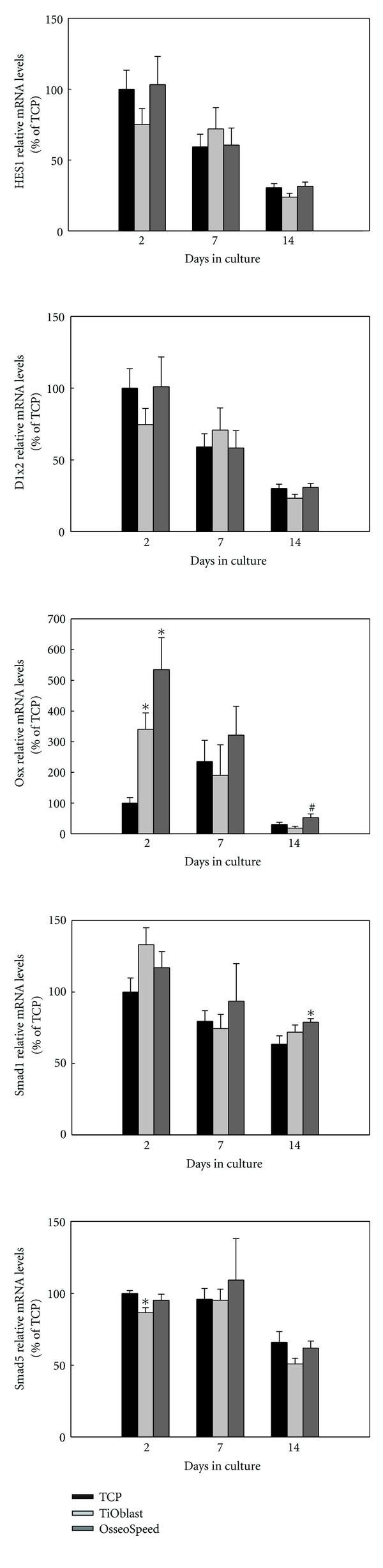
Relative mRNA levels of transcriptional factors and regulators (Hes1, Dlx2, Osx, Smad1, Smad5) after 2, 7 and 14 days of culture. Ratios of target genes relative to housekeeping genes (GAPDH, B-actin) were expressed relative to the mean value of the TCP group at day 2, which was set as 100%. Values represent the mean ± SEM. Differences between groups (*n* = 6) were assessed by Student's *t*-test. ^#^ *P* ≤ 0.05 versus TiOblast; *P ≤ 0.05 versus TCP.

**Figure 8:**
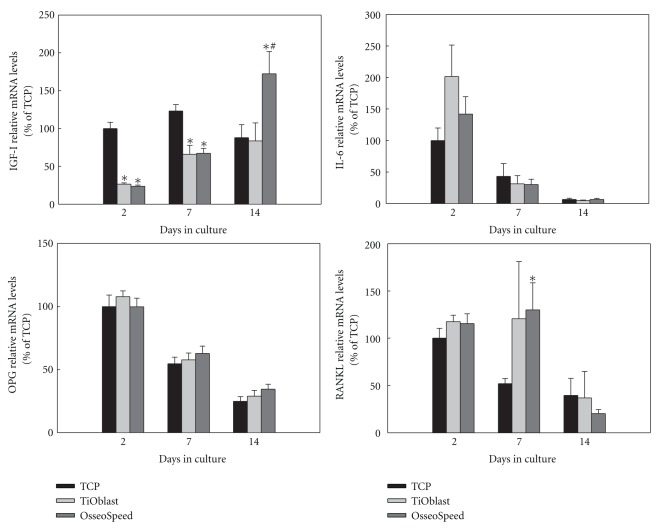
Relative mRNA levels of cytokines and growth factors (IGF-I, IL-6, OPG, RANKL) after 2, 7, and 14 days of culture. Ratios of target genes relative to housekeeping genes (GAPDH, B-actin) were expressed relative to the mean value of the TCP group at day 2, which was set as 100%. Values represent the mean ± SEM. Differences between groups (*n* = 6) were assessed by Student's *t*-test. ^#^ *P* ≤ 0.05 versus TiOblast; *P ≤ 0.05 versus TCP.

The differentiation of MC3T3-E1 cells cultured on the different implant surfaces and onto plastic was examined by analysing the gene expression after 2, 7, and 14 days of cell culture. As seen in Figures 6, 7, and 8, gene expression levels of fifteen different genes related to osteogenic differentiation like transcriptional factors and regulators, extracellular matrix molecules, cytokines, growth factors, and functional markers were analysed. 

Regarding the osteogenic markers (Figure 6), coll-I mRNA expression was significantly higher in cells cultured onto both titanium surfaces compared to those cultured in plastic after 14 days. BSP mRNA expression was upregulated in cells cultured on plastic compared to those cultured on both titanium surfaces. Statistical differences were seen after 2 and 7 days of culture. After 14 days of culture, the OsseoSpeed group displayed higher BSP mRNA levels compared to the TiOblast one. ALP mRNA levels were significantly higher in cells cultured onto plastic compared to those cultured on both titanium surfaces after 2 and 7 days of culture, while after 14 days, ALP levels of cells cultured on both titanium surfaces were higher than those cultured on plastic, although just the OsseoSpeed group reached statistical significance. CD44 mRNA levels were significantly higher in cells cultured on plastic compared to those cultured on both titanium surfaces (day 7) and compared to the TiOblast group (day 2). After 14 days of culture, no differences were observed between groups.

As regards transcriptional factors and regulators (Figure 7), no significant differences were found for Dlx2 and Hes1 among the groups at the different time points, although their gene expression decreased likewise over the time period studied. osterix mRNA levels were higher in cells cultured on both titanium surfaces compared to those cultured on plastic after 2 days of culture. After 14 days of culture, OsseoSpeed implants induced an upregulation osterix mRNA compared to TiOblast. After 14 days of culture, the OsseoSpeed group displayed higher Smad1 mRNA levels compared to TCP. Smad5 mRNA expression was significantly up-regulated in the TCP group compared to the TiOblast one after 2 days of culture, and no differences were observed afterwards. 

Regarding cytokines and growth factors (Figure 8), IGF-I mRNA expression was significantly higher in cells cultured on plastic compared to those cultured on both titanium surfaces after 2 and 7 days of culture. However, after 14 days, IGF-I levels of cells cultured on the OsseoSpeed group were significantly higher than for the other groups. No significant differences were found for IL-6 or OPG mRNA levels among the groups at the different time points and their expression decreased likewise over the time period studied. RANKL mRNA levels (day 7) were significantly higher in the OsseoSpeed group compared to the TCP one. No differences were seen for the other groups or time points for this gene. 

### 3.6. Osteocalcin, Osteoprotegerin, and IL-6 Release

**Figure 9:**
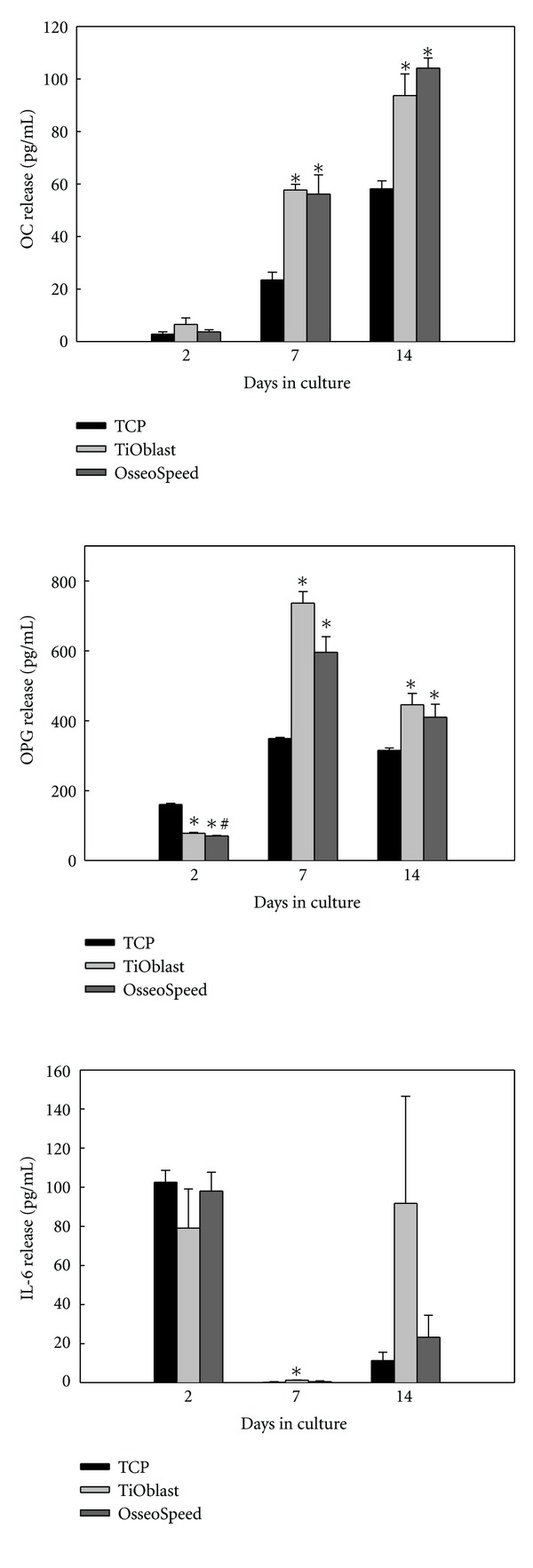
OC, OPG, and IL-6 released to culture media after 2, 7, and 14 days of culture. Values represent the mean ± SEM. Student *t*-test: *P ≤ 0.05 versus TCP; ^#^ *P* ≤ 0.05 versus TiOblast.

Osteocalcin, OPG, and IL-6 release to the culture media was determined by a Luminex bioassay after 2, 7, and 14 days of culture in the TiOblast, the OsseoSpeed and TCP groups (Figure 9). TNF-*α* and RANKL levels were under detection levels in all cell culture supernatants and are thus, not presented. Osteocalcin and OPG release was higher in both implant groups compared to TCP after 7 and 14 days of culture. After 2 days, cells cultured on TiOblast surfaces released higher amounts of OPG than those cultured on OsseoSpeed surfaces. IL-6 release was higher in the TiOblast group than in the TCP one after 7 days of culture. 

### 3.7. ALP Activity and Crystal Deposition

**Figure 10:**
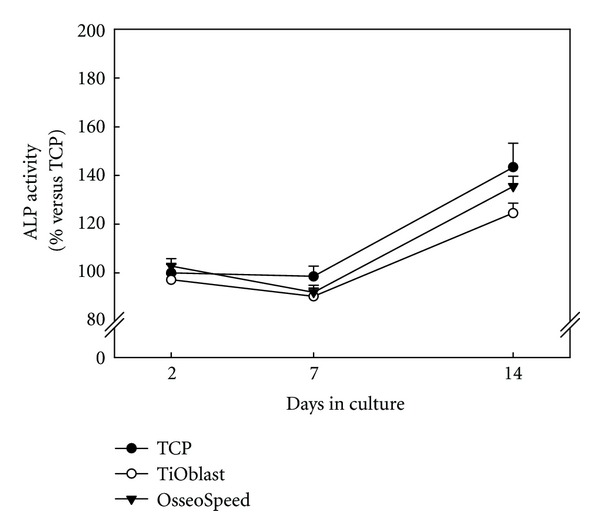
ALP activity measured from culture media collected after 2, 7, and 14 days of culture. Data expressed as a relative to the mean value of the TCP group at day 2, which was set as 100%. Values represent the mean ± SEM. Student *t*-test: *P ≤ 0.05 versus TCP; ^#^ *P* ≤ 0.05 versus TiOblast.

No statistical differences were found for ALP activity in the culture medium after 2, 7, and 14 days of cell culture (Figure 10). However, ALP activity in the OsseoSpeed group tended to be higher than in the TiOblast one. 

After 14 days of culture, cells cultured on OsseoSpeed titanium surfaces displayed a higher number of calcium crystals compared to those cultured on TiOblast surfaces (16.5 ± 1.5 versus 9.5 ± 0.5, respectively; *P* = 0.047). 

## 4. Discussion 

The present study provides a wide characterization of the in vitro osteogenic properties of two commercial surfaces, TiOblast and OsseoSpeed. TiOblast was the first moderately roughened implant surface with 10 years followup reported in the literature and the precursor of the OsseoSpeed surface [18–20]. The OsseoSpeed surface is a further development introduced in 2004 that incorporates small amounts of fluoride ions in the oxide layer, a slight increase on the micrometer scale in surface roughness, and the appearance of a nanoscale topography. In the present work, we showed the topographical differences between the two surfaces, mainly an increase in the *S*_*a*_ value in the OsseoSpeed surface, but also changes in surface skewness, kurtosis, and the core fluid retention index. The obtained results confirm that OsseoSpeed surface show an increased micrometer-scale surface roughness, together with the formation of nanostructures, as reported in earlier studies [11]. The presence of micro- and nanoscale topography in OsseoSpeed compared to TiOblast surface and the addition of fluoride, did not change the biocompatibility of the implants and the initial attachment and proliferation of the MC3T3-E1 cells. However, it was observed that OsseoSpeed surfaces induced a more branched cell morphology. It has been reported that this cell shape may increase the contractility of the cytoskeleton and lead to preferential osteoblastic differentiation [21], which has been found in the present study for the OsseoSpeed surface compared to TiOblast. The increase found in the LDH activity on the TCP surface after 14 days is most probably due to the higher proliferation and/or cellular activity at this later time point on TCP.

MC3T3-E1 osteoblast-like cells undergo a developmental sequence of proliferation and differentiation similar to primary cells in culture [22]. Osteoblast maturation in vitro is characterised by changes in gene expression at each developmental stage [23–26]. Modulation of these expressed genes is subjected to a transcriptional control regulated by growth factors and cytokines [23, 26]. BMP-2 is a highly potent growth/differentiation factor that induces differentiation of progenitor cells into the osteoblast lineage, and exhibits this osteogenic action by activating Smad signaling and by regulating transcription of osteogenic genes. Thus, the higher release of BMP-2 found in the OsseoSpeed group could initiate osteogenic differentiation through the regulation of transcriptional factors.

Runt-related transcription factor 2 (Runx2) is a master regulator of osteogenic gene expression that is necessary for the osteoblast lineage commitment and, as well, regulates osteoblast differentiation [27]. Here, we did not determine Runx2 mRNA levels since, as we have previously reported that Runx2 mRNA expression is constant during osteoblast differentiation [28], probably due to the fact that MC3T3-E1 cells are already committed to the osteoblast lineage. Nevertheless, we have analysed the expression of different transcription factors that have been described to interact with Runx2: Dlx2, a downstream target of BMP-2 that is thought to directly activate Runx2 and Osterix genes [29], and Hes1 that can stimulate the transactivating function of Runx2 [30], although it negatively regulates bone phenotypic maturation and its expression decreases during osteoblast differentiation [31]. We have previously reported that Hes1 and Dlx2 are early responsive genes to roughness and fluoride treatment of titanium implants [32]. In our previous report, both genes were downregulated by fluoride treatment of rough titanium implants after one day in primary human osteoblasts. Here, no differential regulation was found for these two genes among the two surfaces analysed, neither when compared to TCP. The difference between the results may have been caused by differences in the surfaces used for comparison and differences in the cell model. 

The Smad family of proteins has been identified as the downstream propagators of BMP signals [33]. BMP-activated Smads induce Runx2 gene expression and Smads interact physically with the Runx2 protein to induce osteoblast differentiation [34]. In particular, Smad1 and Smad5 are necessary for BMP-mediated Runx2 acetylation [35]. We found no important changes on Smad1 and Smad5 at the different time points and groups analysed; only Smad1 showed higher significant levels in OsseoSpeed compared to TCP. Thus, although Smad expression patterns are informative, future studies should investigate their phosphorylation stage to find out whether their activity is regulated in the different surfaces. 

Osterix is another transcription factor downstream of Runx2 which is required for the ongoing differentiation within the osteogenic pathway [36], being involved in the differentiation step from preosteoblast to fully functional osteoblast [37]. Here we found higher Osx mRNA levels in cells cultured onto OsseoSpeed implants compared to TiOblast, in agreement with earlier observations [8, 10]. 

Other osteogenic markers were analysed during osteoblast differentiation. Type I collagen is expressed in high levels in the early proliferation stage, which is gradually decreased as the cell matures. This downregulation was only observed in the TCP group, while TiOblast and OsseoSpeed showed higher coll-1 mRNA levels. In agreement with these results, Masaki et al. [10] also found higher mRNA levels of coll-I in human palatal mesenchymal stem cells cultured on TiOblast and OsseoSpeed than on TCP after 3 days, although these differences were not significant. Alkaline phosphatase increases during extracellular matrix maturation then decreases when mineralization is well progressed and bone sialoprotein is transiently expressed very early and then upregulated again in differentiated osteoblasts at the onset of mineralization [23, 26]. These two markers, that reflect a more advanced stage of osteoblast differentiation, showed higher mRNA levels on TCP up to the first week, while OsseoSpeed surfaces increased their levels greatly after 14 days, indicating that extracellular matrix of cells seeded on TCP was mature and competent for mineralization after 1 week and on OsseoSpeed surfaces after 2 weeks. This also indicates that differentiation of MC3T3-E1 cells on TiOblast was delayed compared to OsseoSpeed surface. These results are in line with those obtained in the present study for the higher ALP activity and number of crystals deposited in the cell monolayer of OsseoSpeed surface. Using the same in vitro model, another study [8] did not observe significant differences in BSP between the two titanium surfaces, although in this case the roughness was similar, and the titanium particles for grit-blastingwas smaller. Finally, CD44 was analysed as this marker has been indicated to be expressed in higher levels in osteocytes [38]. However, the analysis did not reveal important differences between the two titanium surfaces investigated, only for the TCP group.

Besides these bone-specific markers, the effect of the different titanium implant surfaces on the expression of different growth factors and cytokines involved in bone formation was analysed. IL-6 is a cytokine produced by cells of the osteoblast and osteoclast lineages that not only has a role in inflammation but also increases bone resorption and possibly bone remodeling [39]. Both the mRNA levels and the secretion of IL-6 decreased over the time in cell culture, but in a lesser extent for TiOblast surface. IGF-I induces osteoblast proliferation, bone collagen, and matrix synthesis [40, 41] and stimulates the activity of alkaline phosphatase [42]. Similar to other osteogenic markers commented before, this growth factor was significantly upregulated in OsseoSpeed surface compared to both TCP and TiOblast. Similar results have been found in a previous study in vivo [16], suggesting that IGF-I might play an important role stimulating bone formation when administered in combination with fluoride [43]. 

Osteoblasts exert a crucial function in osteoclast activation and differentiation, through the production of specific biological mediators such as the activator of nuclear factor k B ligand (RANKL) and its antagonist osteoprotegerin (OPG). In the present work, the total amount of RANKL produced by MC3T3-E1 osteoblasts was below the detection limit of the Luminex assay (3 pg/mL), while OPG was detected in the supernatant during the whole culture period, was similar to the reported results by Guida and coworkers using ELISA [44]. However, OPG production found in this study was similar between TiOblast and OsseoSpeed group in MC3T3-E1 osteoblasts, with higher levels on titanium when compared to TCP. As a trend, TiOblast surfaces showed higher levels than OsseoSpeed, opposite to the results obtained in human bone marrow mesenchymal stem cells [44]. Due to the lack of differences in OPG/RANKL mRNA levels and the secretion of OPG, we conclude that in MC3T3-E1 cells, the levels of OPG/RANKL are not regulated by the different surfaces used in the study. Other authors have reported increased OPG levels in response to rough surfaces [45] and different chemical composition [46]. 

In conclusion, the results from the present study demonstrate that the gain of using OsseoSpeed surface is an improved osteoblast differentiation and mineralization, without additional effects on cell viability or proliferation. The enhanced in vitro osteogenic properties are in line with the improved osseointegrating properties and clinical performance of fluoride-modified titanium implants [47–49]. 

## Figures and Tables

**Table 1:** Sense (S) and antisense (A) sequences of the primers used in the real-time PCR of target and housekeeping genes.

Gene	Primer sequence (5′ to 3′)		GeneBank accession number	Amplicon size (base pairs)
ALP	S	AACCCAGACACAAGCATTCC	X13409	151
	A	GAGAGCGAAGGGTCAGTCAG		
BSP	S	GAAAATGGAGACGGCGATAG	L20232	141
	A	ACCCGAGAGTGTGGAAAGTG		
CD44	S	CTTCCATCTTGACCCGTTGT	XM_283773	175
	A	ACAGTGCTCCTGTCCCTGAT		
Coll-I	S	AGAGCATGACCGATGGATTC	NM_007742	177
	A	CCTTCTTGAGGTTGCCAGTC		
Dlx2	S	AGTTCGTCTCCGGTCAACAA	NM_010054.1	125
	A	GCCGCCAGCTGGAAACTGGA		
Hes1	S	CTGCAGCGGGCGCAGATGAC	NM_008235.2	114
	A	ACACGTGGACAGGAAGCGGG		
IGF-I	S	GCTCTTCAGTTCGTGTGTGG	U75390	142
	A	ACATCTCCAGCCTCCTCAGA		
IL-6	S	CCGGGAGCAGTGTGAGCTTA	NM_031168	171
	A	TAGATGCGTTTGTAGGCGGTC		
OPG	S	AGACCATGAGGTTCCTGCAC	U94331	131
	A	AAACAGCCCAGTGACCATTC		
Osx	S	ACTGGCTAGGTGGTGGTCAG	NM_007419	135
	A	GGTAGGGAGCTGGGTTAAGG		
RANKL	S	GGCCACAGCGCTTCTCAG	AF019048	141
	A	TGACTTTATGGGAACCCGAT		
Smad1	S	ATGCCAGCTGACACACCCCC	NM_008539.3	112
	A	TTTCAGCGGGCAGTGGAGGC		
Smad5	S	GGAGTTTGCTCAGCTTCTGG	NM_008541.2	134
	A	TGGTGACGTCCTGTCGGTGGT		

18S rRNA	S	GTAACCCGTTGAACCCCATT	X00686	151
	A	CCATCCAATCGGTAGTAGCG		
GAPDH	S	ACCCAGAAGACTGTGGATGG	XM_132897	171
	A	CACATTGGGGGTAGGAACAC		

**Table 2:** Topographical parameters analysed by blue light profilometry of the titanium surfaces used in the studies.

	TiOblast	OsseoSpeed	*P*
*S*_ a_/*μ*m	0.77 ± 0.06	1.35 ± 0.04	8.3*E* − 08
*S*_ sk_	−0.38 ± 0.05	−0.2 ± 0.08	0.003
*S*_ ku_	3.92 ± 0.05	3.46 ± 0.11	0.00002
*S*_ ci_	1.41 ± 0.02	1.47 ± 0.02	0.002

Mean values and standard deviation are presented (*n* = 5, 3 combined measurements per sample); Student's *t*-test being performed between “TiOblast” and “OsseoSpeed”. Legends: *S*_a_: average height deviation from the mean plan, *S*_sk_: surface skewness, *S*_ku_: surface kurtosis, and *S*_ci_: core fluid retention index.
